# Impact of Quantisal^®^ Oral Fluid Collection Device on Drug Stability

**DOI:** 10.3389/ftox.2021.670656

**Published:** 2021-07-05

**Authors:** Michela Riggio, Keyur A. Dave, Branko Koscak, Mark Blakey, Charles Appleton

**Affiliations:** Central Laboratories, Toxicology, Biochemistry Queensland Medical Laboratory (QML) Pathology, Brisbane, QLD, Australia

**Keywords:** oral fluid, drugs of abuse, collection device, quantisal, mass spectrometry

## Abstract

The stability of drugs can affect drug tests and interpretations. A comprehensive study to verify drug stability in Quantisal^®^ oral fluid (OF) collection device was undertaken in accordance with Australian standard, AS/NZS 4760:2019 (SAI-Global, 2019). The evaluation was performed for the following drugs: (±) amphetamine, (±) methylamphetamine, (±) 3,4-methylenedioxymethylamphetamine (MDMA), (−)Δ9-tetrahydrocannabinol (THC), cocaine, benzoylecgonine, morphine, codeine, and oxycodone. Stability was assessed at four different storage temperatures over seven time points at ±50% cut-off concentrations (Appendix A, Para A4-4.1, AS/NZS 4760:2019) (SAI-Global, 2019). All drugs were found to be significantly more stable at 4 and –20°C, with stability spanning at least 14 days with percentage change within ±20% from the cut-off concentrations (SAI-Global, 2019). In addition, we report a variation trend with cocaine and benzoylecgonine at elevated temperatures, suggesting hydrolytic decomposition of cocaine and a concomitant increase in benzoylecgonine quantitative values. We confirm the cross-talk by showing that the percentage change in the profile of average cocaine-benzoylecgonine measurement is within the acceptance concentration range of ±20%. This finding highlights the importance of precaution during storage and careful considerations during subsequent interpretation of liquid chromatography-mass spectrometry (LCMS) measurements.

## 1. Introduction

Oral fluid (OF), as an alternative, non-invasive, and accessible matrix (Dams et al., 2007; Bosker and Huestis, 2009) for the detection and monitoring of drugs, is of growing interest in clinical toxicology, criminal justice, workplace testing, and driving under the influence of drugs (DUID) programs (Bosker and Huestis, 2009; Zheng et al., 2020). Due to its accessibility, a growing list of illicit drugs are being monitored for quantitative profiling in this matrix (da Cunha et al., 2020) by LCMS analysis; however, it has some pitfalls (Huestis et al., 2011; Desrosiers and Huestis, 2019). Despite its strengths, there are several considerations that any toxicology laboratory must contend to and account for to prevent the pitfalls inherent in analyzing DOA in OF as a matrix:

- Paucity of collection volume and drug concentrations at the analytical limits.
- Shorter detection time frames subsequent to cannabis use is an important consideration during interpretation and test set-up.
- Chemical and physiological factors influencing drug kinetics, disposition, metabolic patterns, and potential contamination.
- Binding and consequential loss of ionized drugs to proteins.
- Relative to other matrices, such as urine, OF can be more infectious due to the presence of more cellular materials.

Consequently, understanding temporal analyte stability and integrity during transportation and specimen storage at relevant temperatures in drug testing laboratories is critical to ensure accurate result interpretation for clinical and forensic purposes (Crouch, 2005; Zaitsu et al., 2008; Ventura et al., 2009; Marchei et al., 2020). In accordance with AS/NZS 4760:2019 (Procedure for specimen collection and the detection and quantification of drugs in OF, Appendix C, Para C4) (SAI-Global, 2019), a comprehensive suitability assessment study of the impact of Quantisal^®^ oral fluid collection device (IMMUNALYSIS™) on drug stability was performed.

Drug stability in fortified human OF was monitored by assessing recovery of the drugs at ±50% cut-offs (SAI-Global, 2019) in Quantisal^®^ OF collection device by quantitative liquid chromatography-mass spectrometry in multiple reaction monitoring (LCMS-MRM) mode of targeted analysis (Supplementary Table 1).

## 2. Materials and Methods

### 2.1. OF Collection Devices and Negative OF Matrix

Quantisal^®^ OF collection tubes were provided by Abbott Rapid Diagnostics containing 3 ml of preservative buffer (final specimen volume after spike: 4 ml). All tubes with preservative buffer were stored in dark at room temperature before the suitability assessment study was carried out. Negative human OF matrix (human saliva) was obtained from GoldenWest Diagnostics, LLC, CA (PM Separations pty ltd., catalog number: OH1060-DF, lot number: E060104), free of psychoactive drugs absence of psychoactive drugs or interferences at the same nominal mass was confirmed by LCMS analysis of the negative matrix as a “patient blank” sample was analyzed concurrently during this study.

### 2.2. Standards and Compounds

All reagents were of analytical or LCMS grade. All organic solvents (methanol and acetonitrile) and ion-pairing agents (formic acid) were LCMS grade (Optima^®^ LCMS grade, Fisher Chemicals). Ultrapure type I water was used for all sample preparations. All deuterated internal standards and certified reference material solutions were obtained from Cerilliant, Round Rock, Texas, USA or Lipomed, Cambridge, MA, USA (For lot numbers and expiry refer Supplementary Tables 1–3) at 1 mg/ml and 100 μg/ml concentrations. The stock solutions were diluted with LCMS grade methanol to obtain intermediate stock concentrations. Periodically calibrated, cleaned, and internally verified positive air displacement pipettes were used for all sample preparation.

### 2.3. Analytical Method

All procedures were performed in accordance with Australasian standards, standard operating procedures, and validated methods. Human OF (neat) was fortified at two different analytical cut-offs as outlined in “Procedure for specimen collection and the detection and quantification of drugs in oral fluid” (Appendix C, Para C4, AS/NZS 4760:2019) (SAI-Global, 2019). The verification was performed with 20 OF collection devices (*n* = 20) for each of the concentrations outlined in Supplementary Table 1, following the guidelines (SAI-Global, 2019) stated below:

1. Below the cut-off to no more than –50% of the cut-off.
2. Above the cut-off to no more than +50% of the cut-off.

Fortified human oral fluids at the above concentrations were prepared in a volumetric flask and added in to Quantisal^®^ OF collection tubes. Sample preparation and drug extractions were performed using standard operating procedures (QML Pathology SOP number SOP/BI/06/25) for confirmation of drugs of abuse in OF, validated in accordance with the guidelines of the AAFS Standards Board (ASB), (AAFS Standards Board, 2019) formerly known as Scientific Working Group for Forensic Toxicology (SWGTOX), standard practices for the validation of the method used in forensic toxicology (Toxicology, 2013). Briefly, fortified neat human OF samples were diluted 1:8 times in 20% (v/v) methanol containing 2% (v/v) formic acid. Calibrators were spiked in the same manner as neat human OF. Internal standards were spiked at the same level in both fortified neat human OF and calibrator samples. All samples were vortexed, centrifuged, transferred to glass vials, and analyzed by LCMS.

Liquid chromatography-mass spectrometry analysis was performed using SCIEX ExionLC^®^ interfaced to 6500+ QqQ triple quadrupole MS. Chromatographic separation was carried out using 50. mm x 3. mm, 2.6μm, 100Å, Kinetex^®^ Biphenyl column coupled with SecurityGuard^®^ ULTRA cartridge of the same chemistry. Two scheduled MRM transitions (quantifier and qualifier) were recorded for each of the drug standards and one for the corresponding internal standards. A fresh seven-point standard curve (2.5–250 ng/ml) was prepared and analyzed after every 40 samples at each time point. Quality control samples, prepared from a different source of certified reference material at ±40% of the mandated confirmatory test cut-off concentrations (SAI-Global, 2019), were also measured by LCMS-MRM to ascertain accuracy of the standard curve.

### 2.4. Study Design

Fortified human OF at ±50% cut-off concentrations in Quantisal^®^ OF collection tubes was stored at different storage temperatures and were extracted on different days as summarized in Table 1. If the same collection device stored at –20°C is used to study the stability of drugs, the freeze-thaw cycles required at each time point would result in drug losses. To preclude confounding technical variance resulting from the freeze-thaw cycles and concomitant degradation, separate collection devices were assigned to each day of the time point, only to be taken out of storage and extracted on designated days.

**Table 1:** Study design.

**Drug Standards (cut-offs)**	**Storage temperature**	**Days**
–50%	37°C (water bath)	1, 2, 3, 5, 7, 14, 30
	RT (~24°C)	1, 2, 3, 5, 7, 14, 30
	4°C (Refrigeration)	1, 2, 3, 5, 7, 14, 30
	–20°C (Freezer)	1, 2, 3, 5, 7, 14, 30
+50%	37°C (water bath)	1, 2, 3, 5, 7, 14, 30
	RT (~24°C)	1, 2, 3, 5, 7, 14, 30
	4°C (Refrigeration)	1, 2, 3, 5, 7, 14, 30
	–20°C (Freezer)	1, 2, 3, 5, 7, 14, 30

### 2.5. Data analysis

Post-acquisition analysis and peak integration were performed using MultiQuant^™^(SCIEX, version 3.0.3) software, and the concentrations for each of the drugs were obtained by a linear regression analysis using the analyte/internal standard area ratio vs. analyte concentration.

Percent change relative to the stipulated cut-offs (±50%, Supplementary Table 1) (SAI-Global, 2019) was calculated for each time point across all drugs at both concentrations. The drug was considered to be stable at the specified temperature and time point if the percentage difference of the average concentration of the drug (*n* = 20) did not deviate by more than ±20% of the cut-off (Supplementary Table 1) (SAI-Global, 2019). Percentage change was calculated as follows:


(1) $$\begin{array}{l} (\%)=\left(\frac{v1-v2}{v2}\right)\times 100 \end{array}$$

where *v*1 is the observed average concentration of the time course samples and *v*2 is the stipulated cut-off (Supplementary Table 1).

Data extraction, analysis, and visualization were conducted in R (R statistical programming language, version 4.0.2), R Core Team (2020).

## 3. Results and Discussion

Human OF was pooled and centrifuged, and the supernatant used for subsequent spiking of the required drugs was extracted and analyzed by LCMS-MRM. Recoveries at four different temperatures (RT, 37, 4, and −20°C,), two levels of drug concentrations (±50%), and seven time points (1, 2, 3, 5, 7, 14, and 30 days) were assessed. Overall, drugs at both levels were more stable in the Quantisal^®^ buffer over longer time periods at 4°C (**Figures 2**, **3**, Supplementary Figures 5, 6) and –20°C (Supplementary Figures 1–4). It appears that except for Δ9-tetrahydrocannabinol (THC), cocaine, and benzoylecgonine, the buffer solution in Quantisal^®^ helps in preserving stability of all other drug classes with recoveries well within ±20% at room temperature and 37°C for at least 14 days. At room temperature and 37°C, labile drugs, such as cocaine, showed rapid hydrolytic decomposition after 24 h (at 37°C) with a concomitant increase in benzoylecgonine (**Figure 4**), as reported in previous studies (Kiszka et al., 2000; Warner and Norman, 2000; Clauwaert et al., 2004; Duer et al., 2006; Zaitsu et al., 2008; Bijlsma et al., 2013; Bisceglia and Lippa, 2014; D'Elia et al., 2017).

### 3.1. Amphetamine-Type Substances

**Figure 1:**
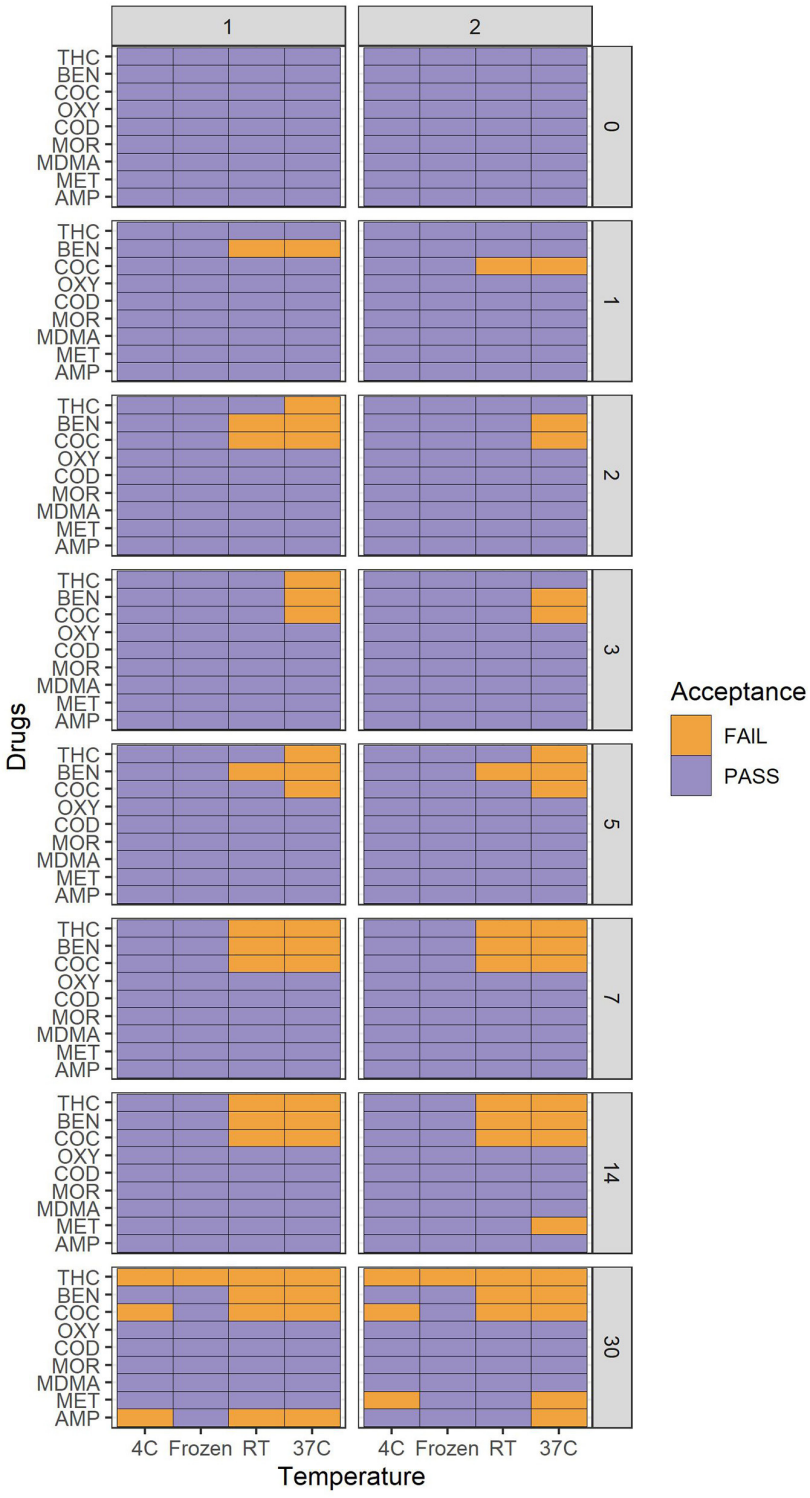
Heatmap of drug stability within ±20% acceptance range over a period of 30 days. Within ±20% = "Pass" or "Fail". The y-axis represents drugs tested, and x-axis represents storage temperature. Vertical facet 1 = −50% and facet 2 = +50% cut-off. Horizontal facets represents days from 0 to 30.

In this drug class racemic mixtures (±) of amphetamine, methylamphetamine, and 3,4- methylenedioxymethylamphetamine (MDMA) were assessed. Recoveries of all three drugs were within ±20% of their target values. The density distribution of calculated concentrations from 20 replicate measurements suggested consistent stability of the analytes in Quantisal^®^ buffer solution for at least 14 days (except methylamphetamine) as shown in Figure 1. A larger variance in measurement was observed at +50% targeted cut-off at 37°C (Supplementary Figures 13, 14), most likely due to the impact of evaporation and subsequent changes in volumes. This technical confounder at elevated temperatures impacted some replicate measurements, and they were observed to skew the calculated mean value away from the central tendency of the distribution, which was found to be well within ±20% in all three drugs of this class (Supplementary Figures 13, 14). This means that, at elevated temperatures, a small proportion of results are likely to produce false negatives due to evaporation-related phenomenon up to 14 days of storage; however, most will produce positive results until this duration.

### 3.2. Cannabinoids

**Figure 2:**
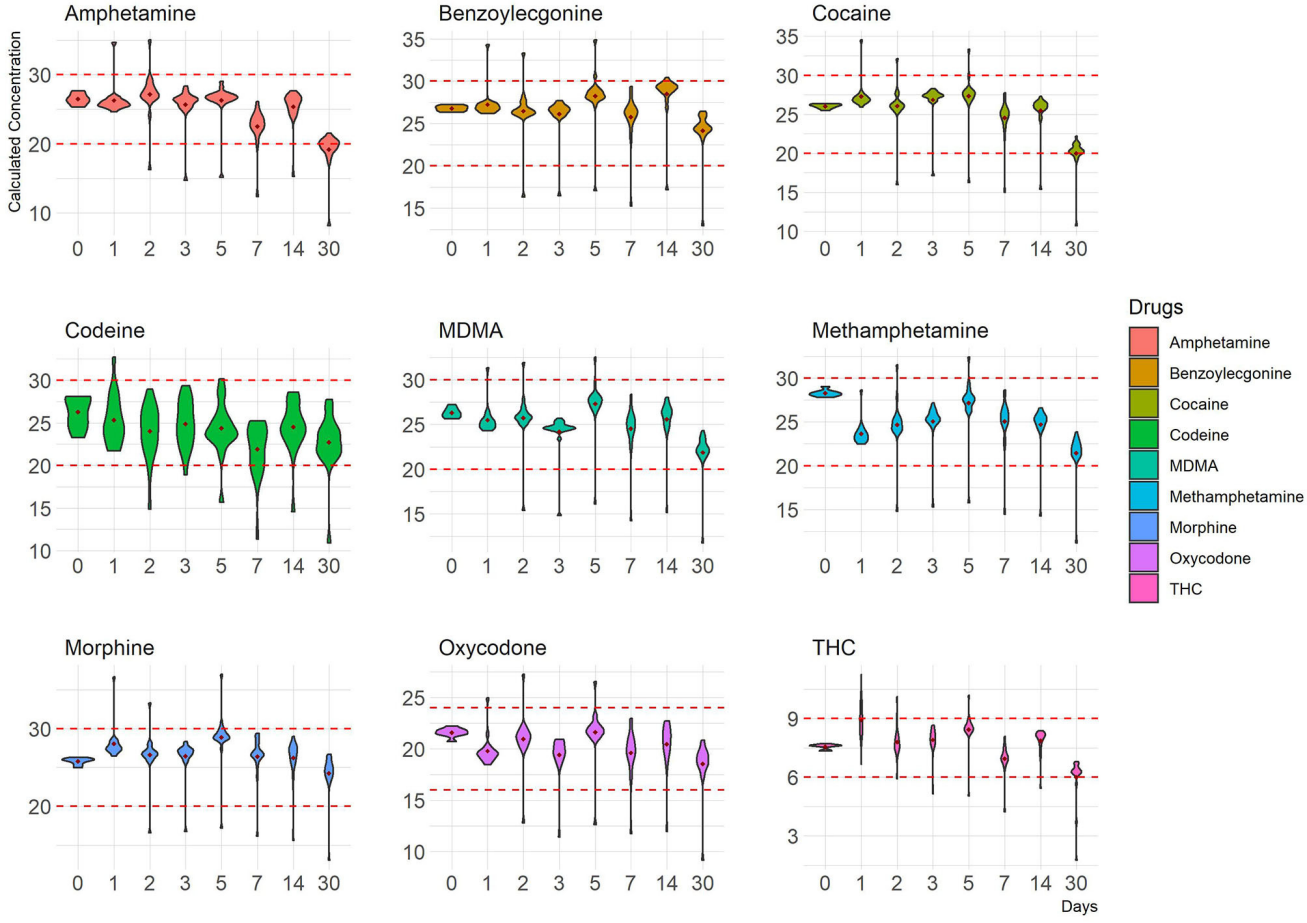
Concentration distribution of replicate measurements (–50% cut-off) of nine drugs at 4°C is depicted as violin plots that outline kernel probability density, i.e., the width of the shaded colored area represents the proportion of the data located there. The y-axis represents calculated concentrations, and the x-axis represents time points in days. Red dotted lines represent ±20% cut-off.

**Figure 3:**
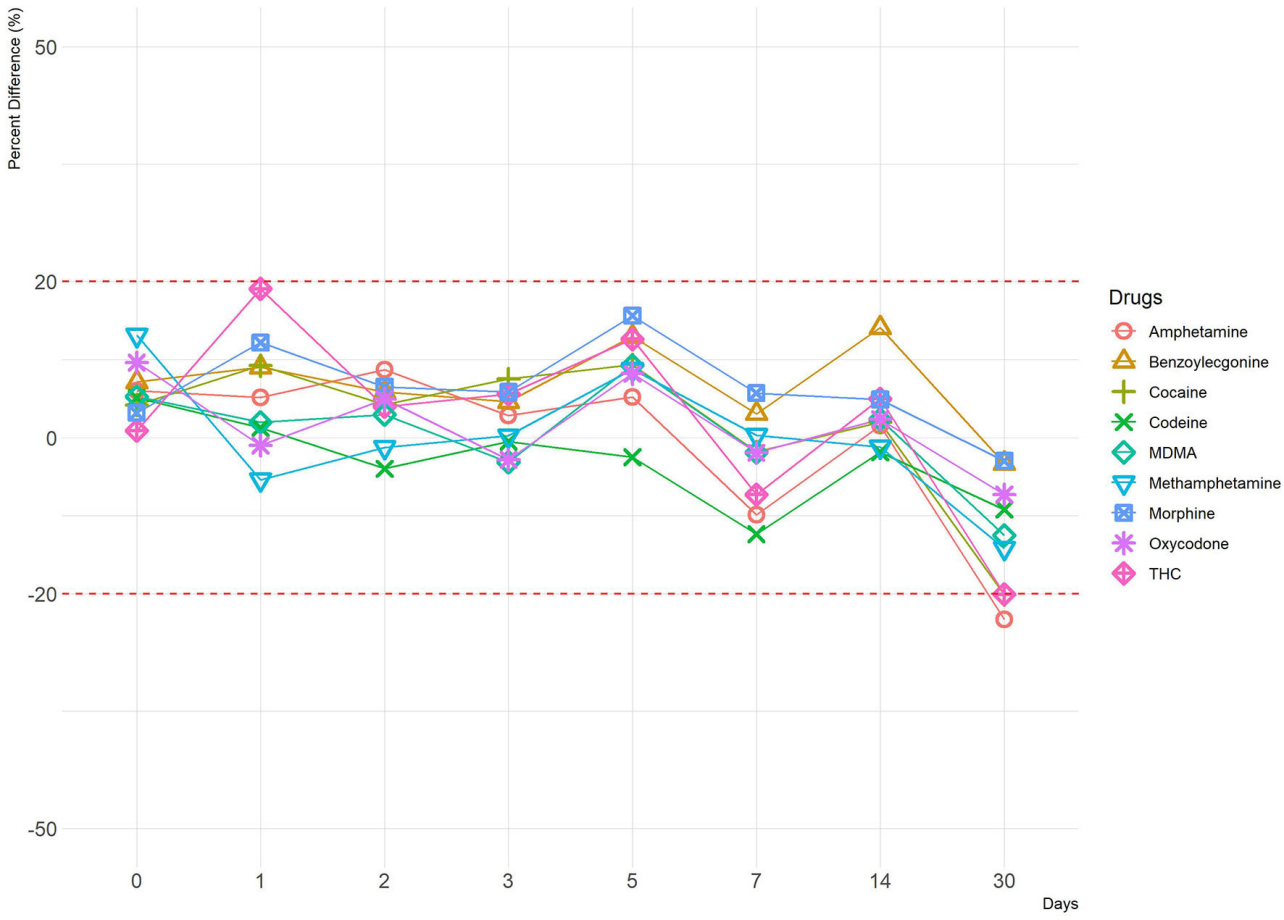
A line plot depicting percentage difference at 4°C (–50% cut-off). The y-axis represents percentage difference, and the x-axis represents time points in days. Red dotted lines represent ±20% cut-off.

In this drug class, Δ9-THC was assessed. Numerous studies have highlighted various aspects of cannabinoid (and metabolites) stability in a variety of matrices and the impact of extraneous factors (pH, temperature, light, etc.) during collection, extraction, and storage (Moore et al., 2007; Lee et al., 2012; Scheidweiler et al., 2017). In general, the spread of measurements for replicates was observed to be larger at –50 than +50% targeted cut-off (Figures 2, 3, Supplementary Figures 1–14). This is congruent with increased stability at higher concentrations of Δ9-THC. Both 4°C and –20°C were observed to be ideal temperatures for extended stability (at least 14 days in Quantisal^®^ buffer solution) as shown in Figures 2, 3, and Supplementary Figures 1–6. On the contrary, RT and 37°C showed faster degradation with stability up to 5 days and at least 24 h, respectively, dependent on spiked concentration (Supplementary Figures 7–14). We recommend that the storage temperature for this class of drug analyzed from OF collected in Quantisal^®^ buffer solution should be preserved at or below 4°C.

### 3.3. Cocaine and Metabolites

**Figure 4:**
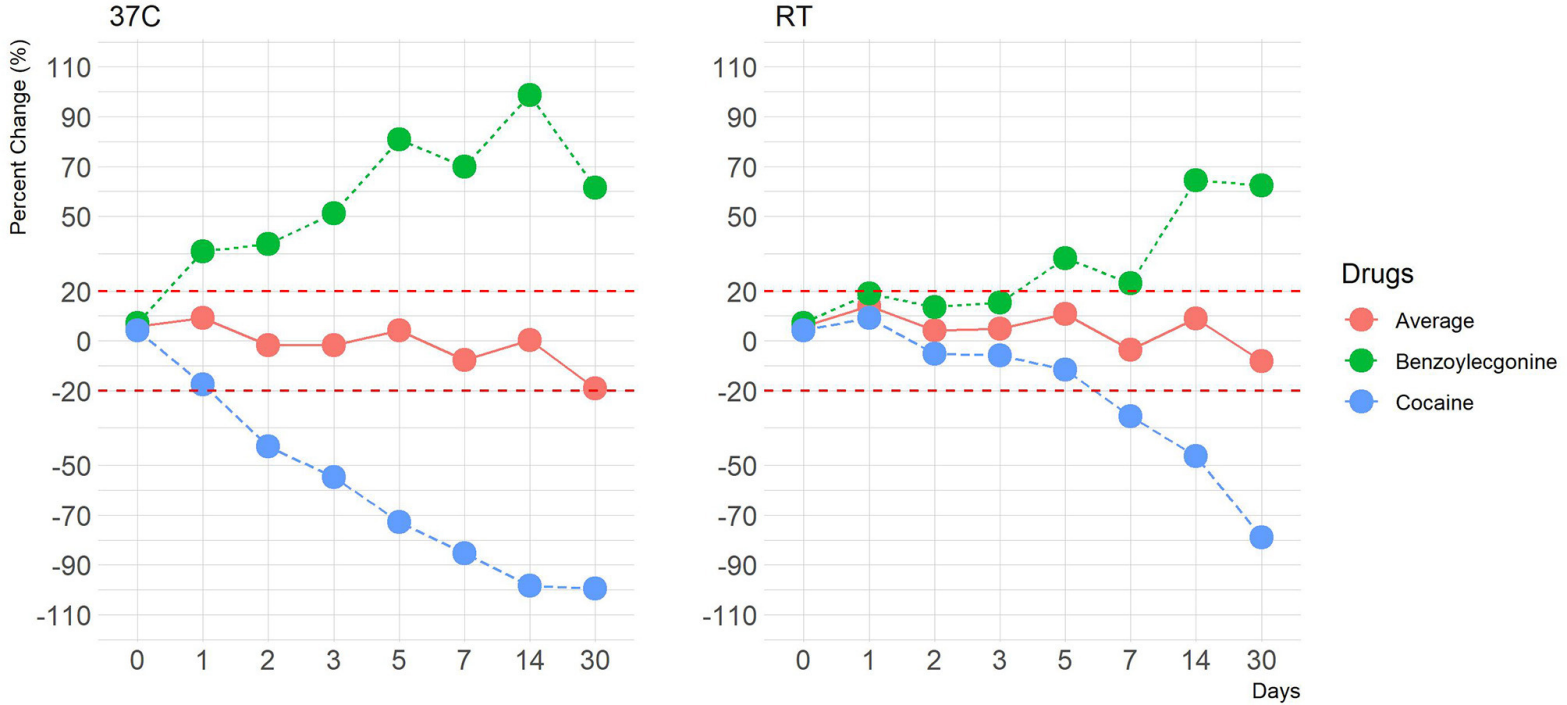
A line plot depicting percentage differences at 37°C and at room temperature (–50% cut-off) of average cocaine and benzoylecgonine concentration values. The y-axis represents percentage change, and the x-axis represents time points in days. Red dotted lines represent ±20% cut-off.

In this drug class, cocaine and benzoylecgonine were assessed. We have observed a variation trend in cocaine and benzoylecgonine at room temperature and at 37°C, which corroborates findings from other studies (Kiszka et al., 2000; Warner and Norman, 2000; Clauwaert et al., 2004; Duer et al., 2006; Zaitsu et al., 2008; Bijlsma et al., 2013; Bisceglia and Lippa, 2014; D'Elia et al., 2017). OF as a matrix, potentially comprises of enzymes and proteins and has been shown to accelerate hydrolytic degradation of cocaine to benzoylecgonine, particularly at elevated temperature and less acidic environment, which we also confirm in this study (Supplementary Figures 7–14) (D'Elia et al., 2017). Consequently, at elevated temperatures, cocaine and benzoylecgonine showed inverse profiles with a marked decrease in the cocaine and a corresponding increase in benzoylecgonine channel outside the acceptable percentage change of ±20%, even as early as after 24 h (day) of incubation. Considering that this is a cross-talk between cocaine and benzoylecgonine, we predicted that mean concentration value of the two drugs and percentage change from the targeted cut-off would be within ±20% of acceptable range. Figure 4 shows this case. Quantisal^®^ buffer solution does not appear to restrict the hydrolytic activity, and considered approach must be taken if collection devices are stored at temperatures exceeding 4°C. For samples stored at elevated temperatures, we propose that cocaine and benzoylecgonine concentrations are interpreted together. This will prevent false negative cocaine results, as benzoylecgonine will produce positive results when confirmed at the laboratory; however, the accuracy of the concentration can be questionable and should always be compared with appropriately stored samples (at or below 4°C).

### 3.4. Opiates

In this drug class, morphine and codeine were assessed. Morphine and codeine were both observed to be consistently within ±20% acceptable range over a period of at least 14 days at all four storage temperatures (Figures 2, 3, Supplementary Figures 1–14). This indicates that Quantisal^®^ buffer solution helps preserve the integrity of this class of drugs over prolonged storage periods, over at least 14 days, at usually encountered temperature conditions during transport. 6-monoacetylmorphine was not included in this study despite being a marker of consumption. This exclusion was premised on transportation storage stability evaluation as per Australia/New Zealand AS/NZS 4760:2019 standards (SAI-Global, 2019), in accordance with the use of the Quantisal^®^ device also for on-site screening.

### 3.5. Oxycodone

In this drug class, oxycodone was assessed. Recoveries as percentage change for oxycodone at both spike concentrations were within ±20% of their target values. Consistent performance was observed across all replicates suggesting effective preservation of oxycodone in Quantisal^®^ buffer solution (Figures 2, 3, Supplementary Figures 1–14).

## 4. Conclusion

This is the most comprehensive evaluation of transport conditions encountered during OF collection by routinely used Quantisal^®^ device, and their suitability was assessed in terms of accuracy and variance in LCMS-MRM measurements of various illicit drugs at four storage temperatures over a period of 30 days with seven time points. We show that, for most classes of drugs (amphetamine-type substances, cannabinoids, cocaine and metabolites, opioids, and oxycodone) the most suitable short-term storage temperature was found to be between 1 and 4°C (Figures 2, 3, Supplementary Figures 1–14). Storage at −20°C would be appropriate for long-term storage (i.e., in cases of a dispute).

Below is a summary of drugs that were found to be unstable with recoveries outside of the acceptable range (±20% change)

1. **Cocaine was stable up to the following period**
  - 7 days (±50% cut-off) at room temperature
  - 2 days (–50% cut-off) and 1 day (+50% cut-off) at 37°C
2. **Benzoylecgonine was stable up to the following period**
  - 5 days (±50% cut-off) at room temperature
  - 1 day (–50% cut-off) and 2 days (+50% cut-off) at 37°C
3. **Δ9-THC was stable up to the following period**
  - 7 days (±50% cut-off) at room temperature
  - 2 days (–50% cut-off) and 5 days (+50% cut-off) at 37°C
4. **Amphetamine was stable up to the following period**
  - 30 days (–50% cut-off) at room temperature
  - 30 days (±50% cut-off) at 37°C
  - 30 days (–50% cut-off) at 4°C
5. **Methylamphetamine was stable up to the following period**
  - 30 days (+50% cut-off) at 37°C
  - 30 days (+50% cut-off) at 4°C

All other drugs were stable within ±20% change from the target value up to and including 30 days as summarized in the Figure 1.

## Data Availability Statement

The original contributions presented in the study are included in the article/Supplementary Material, further inquiries can be directed to the corresponding author/s.

## Author Contributions

MR, KD, BK, MB, and CA contributed to conception, design of the study, and wrote sections of the manuscript. MR performed the experiments. KD performed data analysis and wrote the first draft of the manuscript. All authors contributed to manuscript revision, read, and approved the submitted version.

## Conflict of Interest

The authors were employed by company Queensland Medical Laboratory (QML) Pathology, under the Healius Pathology Pty Ltd network. QML Pathology has a commercial supplier/customer relationship with Abbott Diagnostics for supply of laboratory reagents, analysers, etc as the laboratory does with many other suppliers. Abbott Diagnostics assisted by providing collection and transportation devices and by recompensing the laboratory for cost of consumables required to perform the validation. This had no influence on the performance of the investigation or the conclusions reached.
