# Supplementary material for: Impact of Quantisal® Oral Fluid Collection Device on Drug Stability
Source: Front Toxicol. 2021 Jul 5;3:670656. doi: 10.3389/ftox.2021.670656 (PMC8915805; doi:10.3389/ftox.2021.670656)
Supplement: Supplementary file 1 [file Data_Sheet_1.pdf]

Supplementary Material

1 SUPPLEMENTARY FIGURES

1.1 Figures

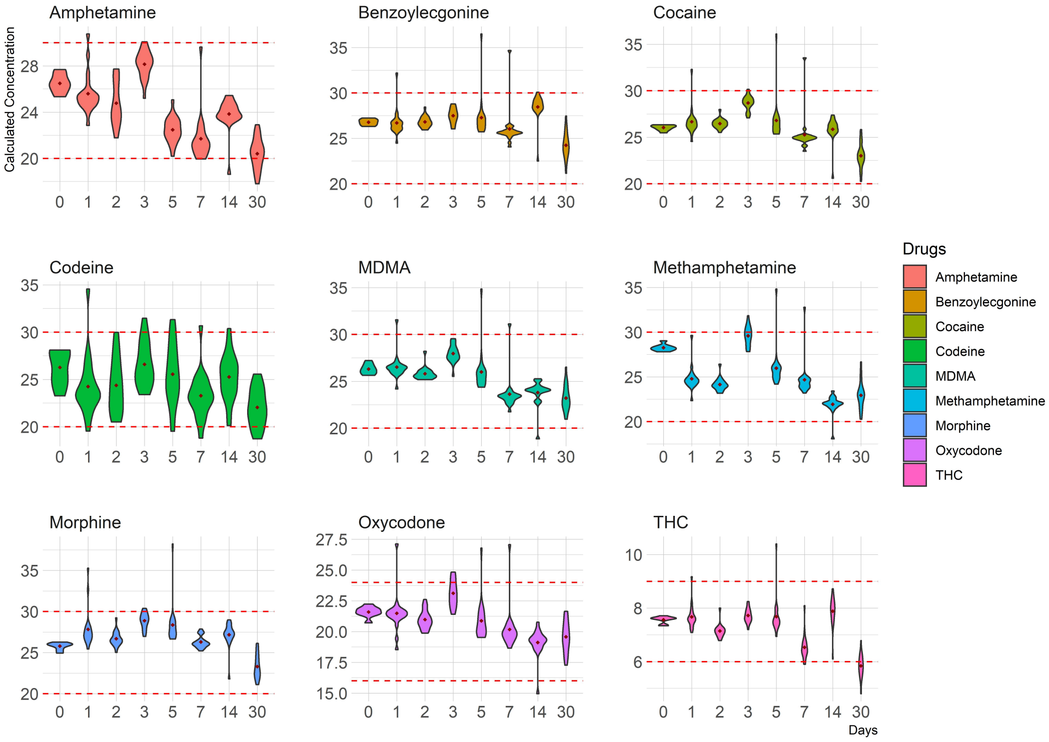

Figure S1. Concentration distribution of replicate measurements (-50% cut-off) of nine drugs at -20°C is depicted as violin plots that outline kernel probability density, i.e. the width of the shaded coloured area represents the proportion of the data located there. The y-axis represents calculated concentrations and x-axis represents time points in days. Red dotted lines represent ±20% cut-off.

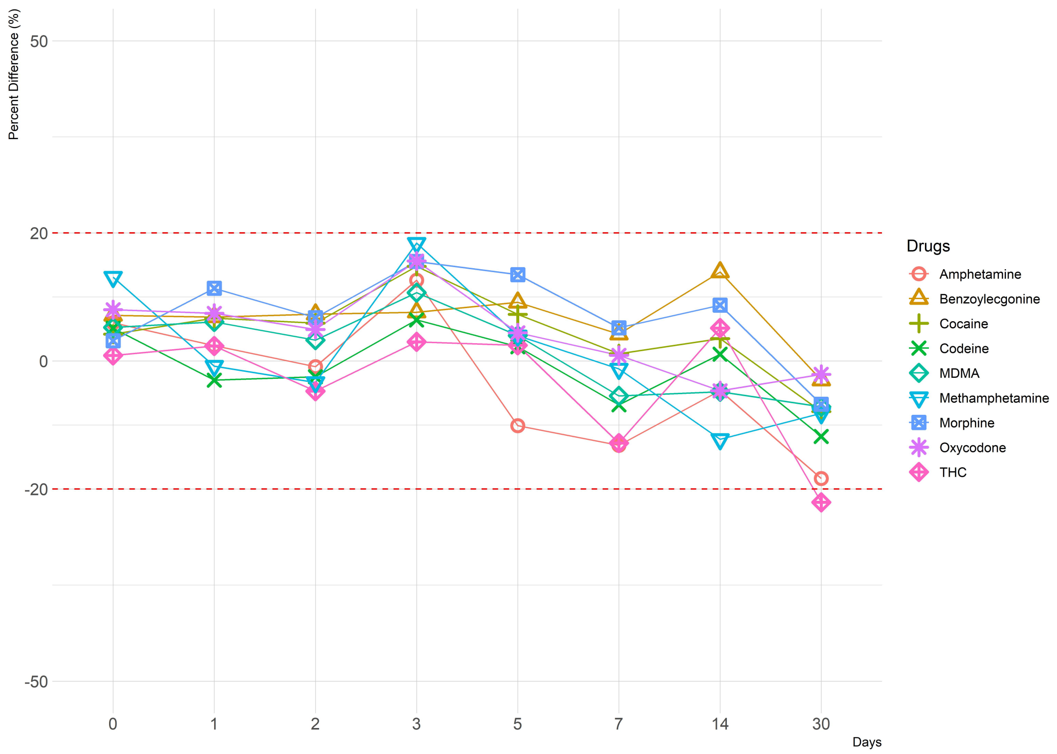

Figure S2. A line plot depicting percentage difference at -20°C (-50% cut-off). The y-axis represents percent difference and x-axis represents time points in days. Red dotted lines represent ±20% cut-off.

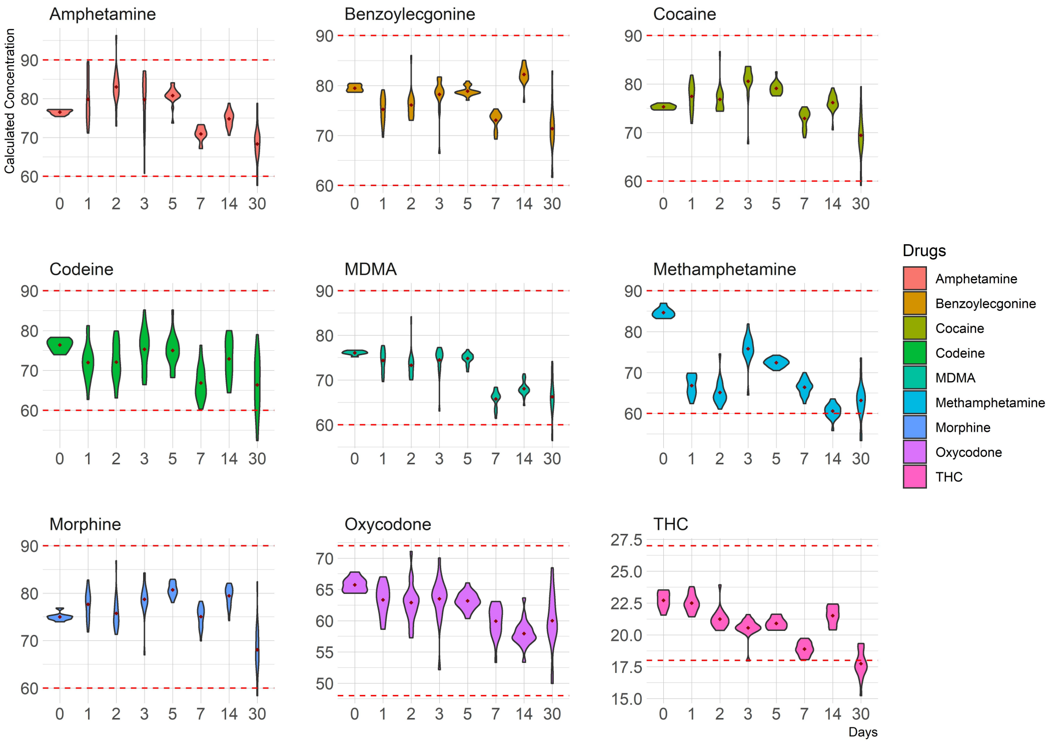

Figure S3. Concentration distribution of replicate measurements (+50% cut-off) of nine drugs at -20°C is depicted as violin plots that outline kernel probability density, i.e. the width of the shaded coloured area represents the proportion of the data located there. The y-axis represents calculated concentrations and x-axis represents time points in days. Red dotted lines represent ±20% cut-off.

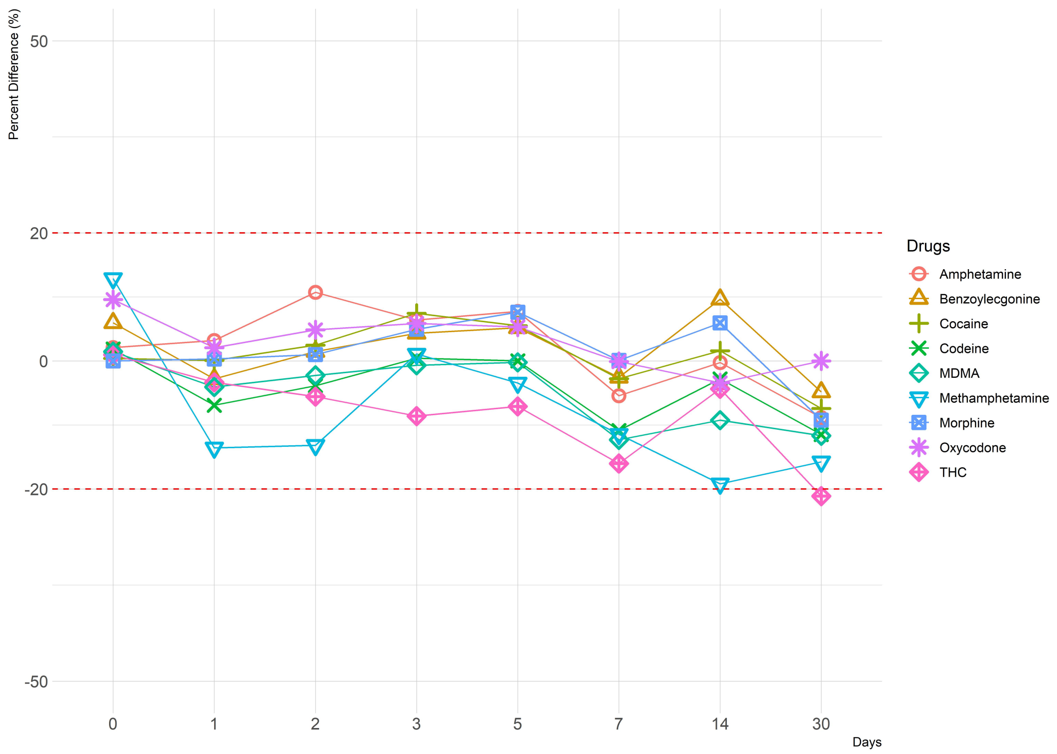

Figure S4. A line plot depicting percentage difference at -20°C (+50% cut-off). The y-axis represents percent difference and x-axis represents time points in days. Red dotted lines represent ±20% cut-off.

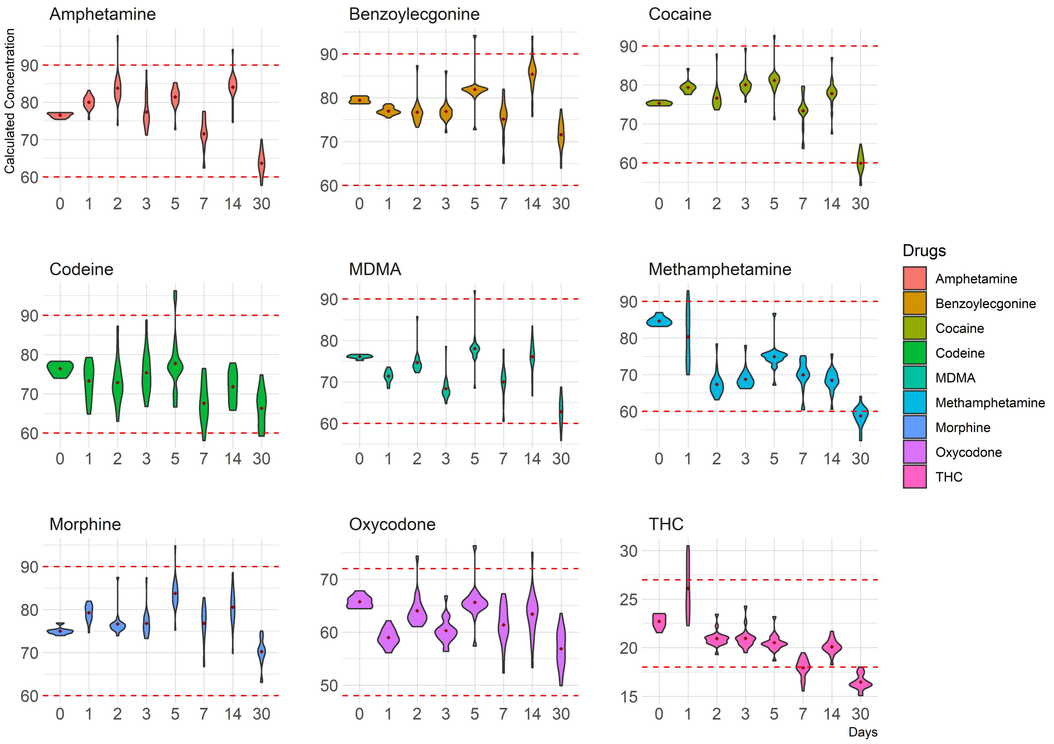

Figure S5. Concentration distribution of replicate measurements (+50% cut-off) of nine drugs at 4°C is depicted as violin plots that outline kernel probability density, i.e. the width of the shaded coloured area represents the proportion of the data located there. The y-axis represents calculated concentrations and x-axis represents time points in days. Red dotted lines represent ±20% cut-off.

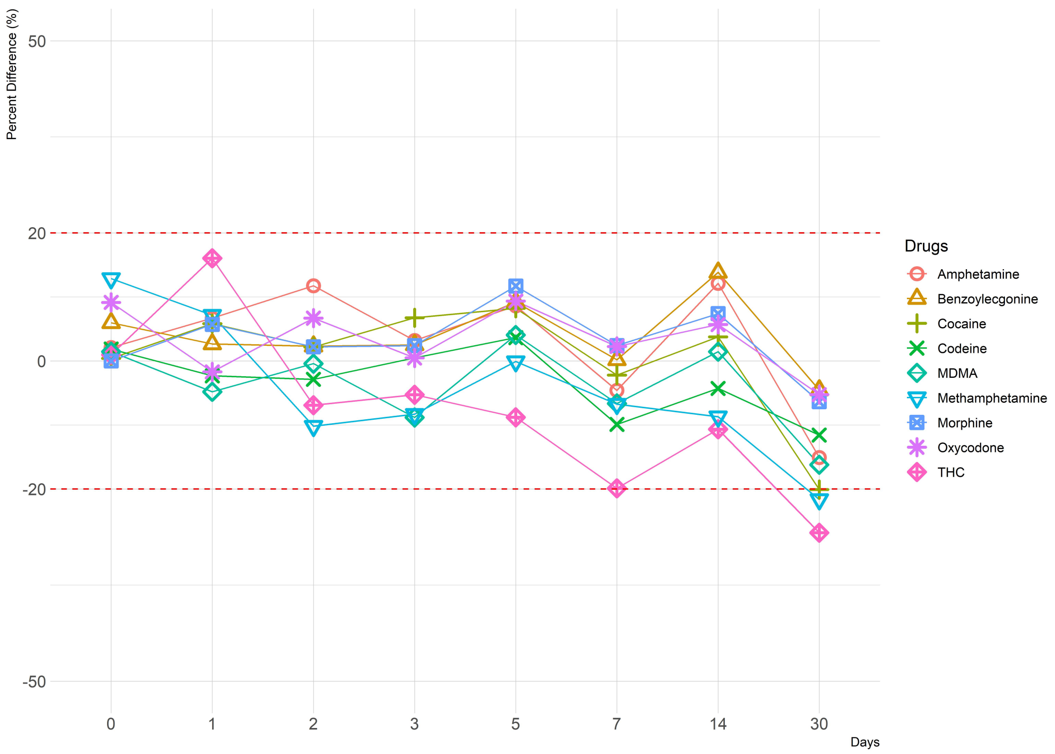

Figure S6. A line plot depicting percentage difference at 4°C (+50% cut-off). The y-axis represents percent difference and x-axis represents time points in days. Red dotted lines represent ±20% cut-off.

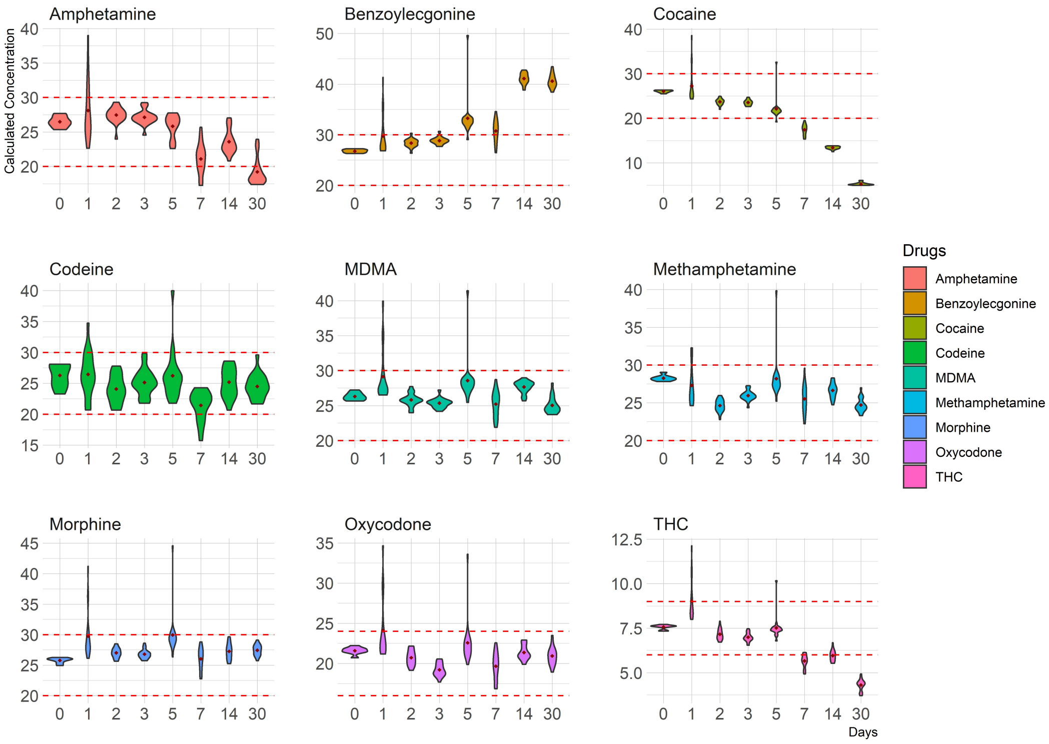

Figure S7. Concentration distribution of replicate measurements (-50% cut-off) of nine drugs at RT is depicted as violin plots that outline kernel probability density, i.e. the width of the shaded coloured area represents the proportion of the data located there. The y-axis represents calculated concentrations and x-axis represents time points in days. Red dotted lines represent ±20% cut-off.

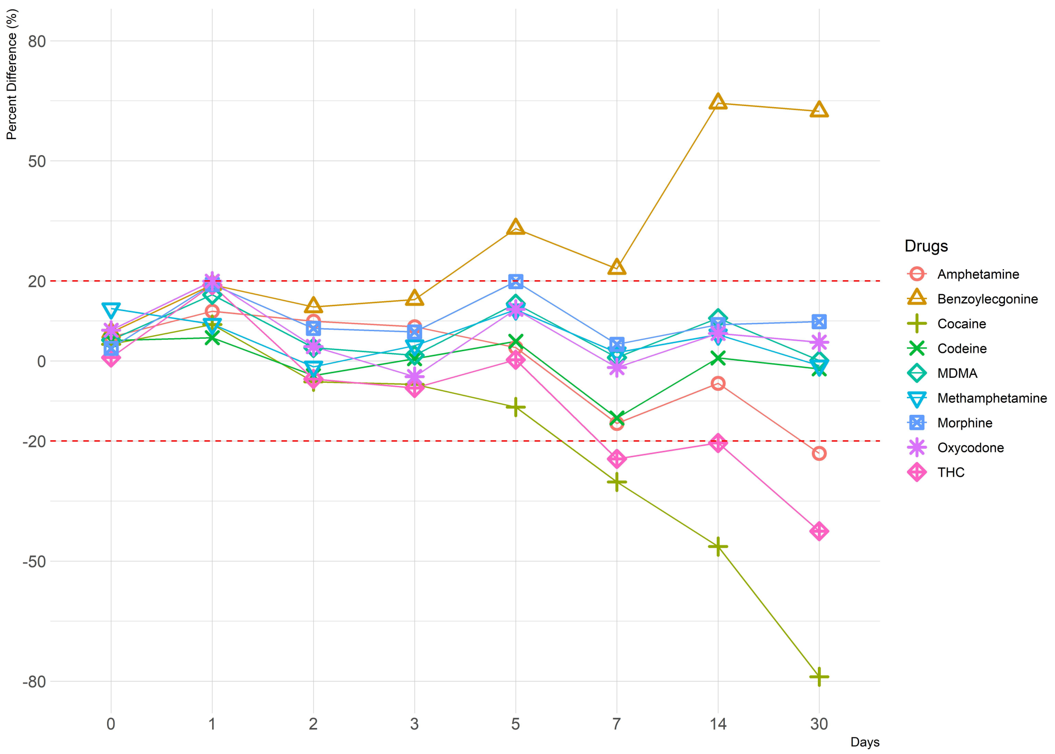

Figure S8. A line plot depicting percentage difference at RT (-50% cut-off). The y-axis represents percent difference and x-axis represents time points in days. Red dotted lines represent ±20% cut-off.

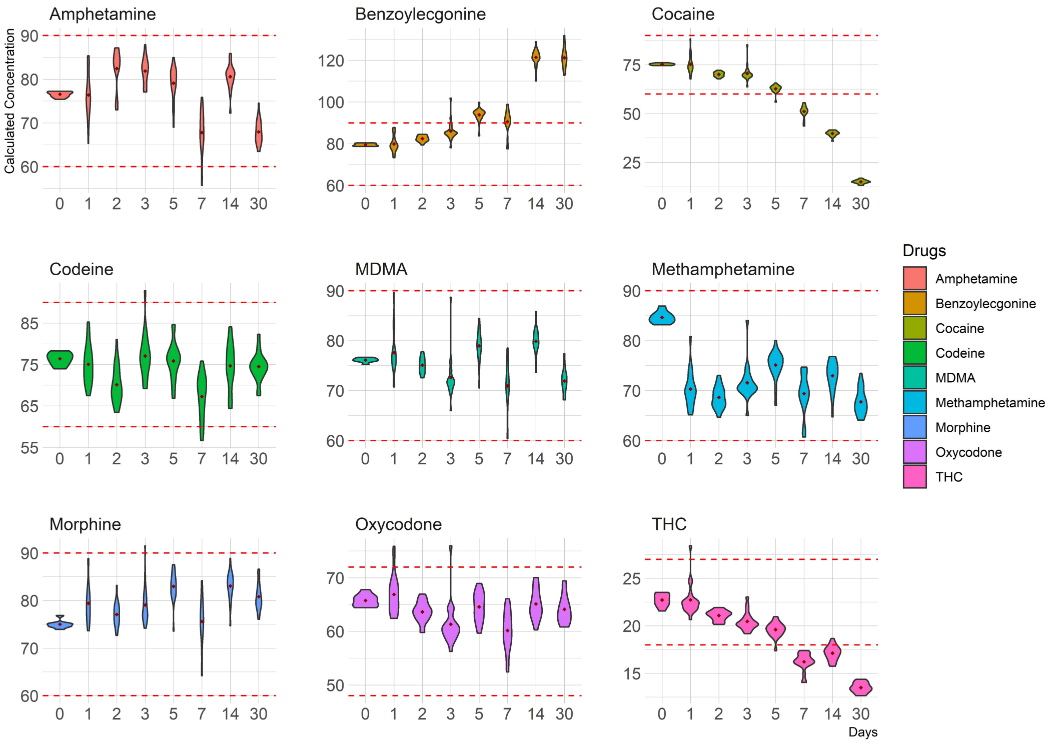

Figure S9. Concentration distribution of replicate measurements (+50% cut-off) of nine drugs at RT is depicted as violin plots that outline kernel probability density, i.e. the width of the shaded coloured area represents the proportion of the data located there. The y-axis represents calculated concentrations and x-axis represents time points in days. Red dotted lines represent ±20% cut-off.

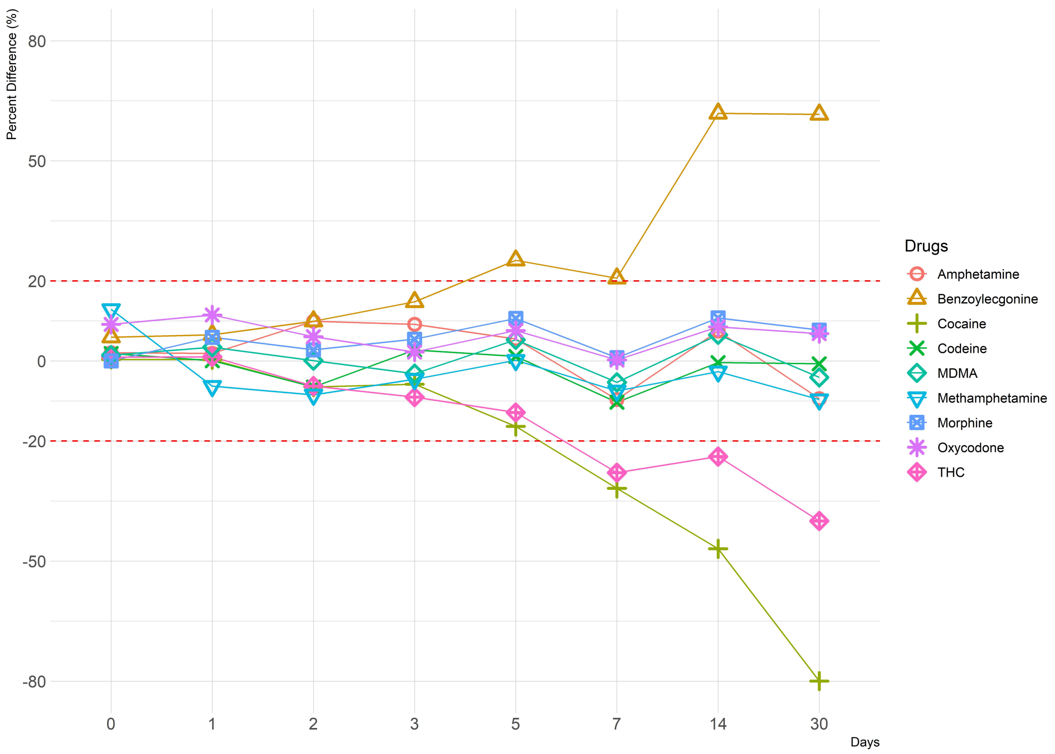

Figure S10. A line plot depicting percentage difference at RT (+50% cut-off). The y-axis represents percent difference and x-axis represents time points in days. Red dotted lines represent ±20% cut-off.

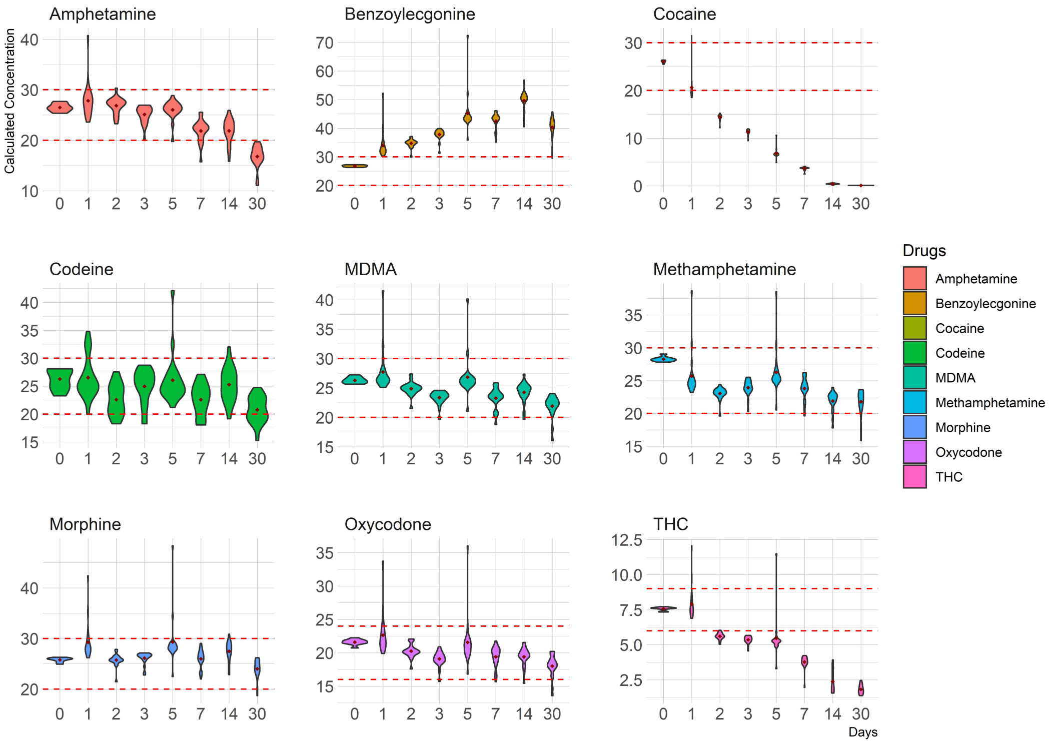

Figure S11. Concentration distribution of replicate measurements (-50% cut-off) of nine drugs at 37°C is depicted as violin plots that outline kernel probability density, i.e. the width of the shaded coloured area represents the proportion of the data located there. The y-axis represents calculated concentrations and x-axis represents time points in days. Red dotted lines represent ±20% cut-off.

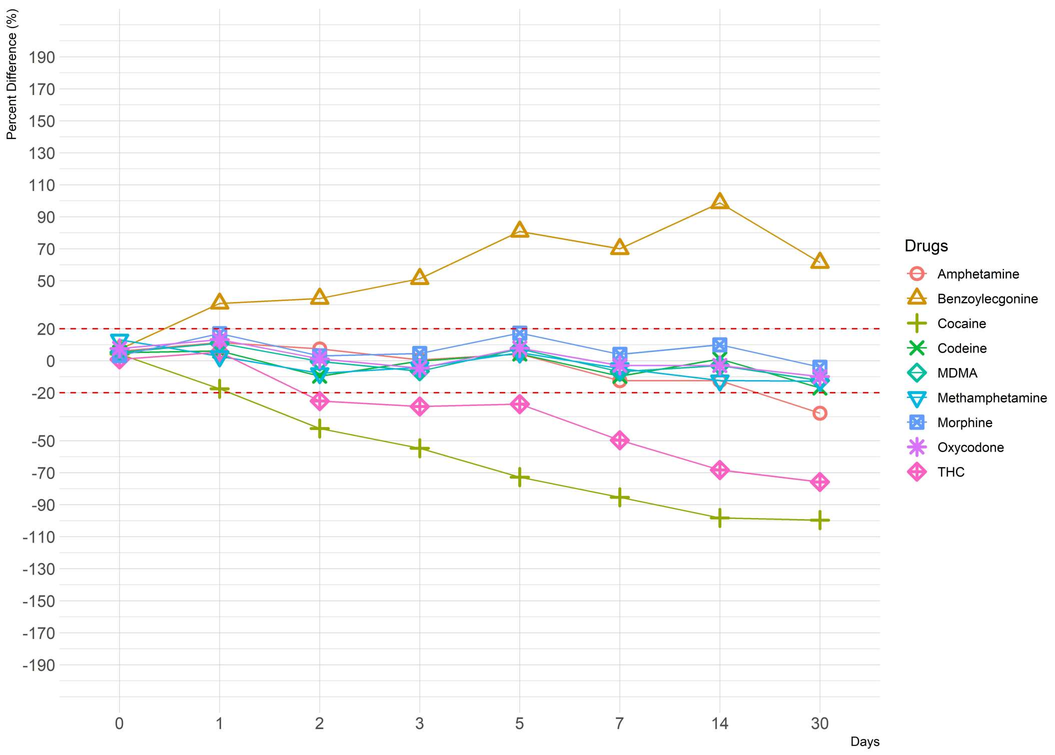

Figure S12. A line plot depicting percentage difference at 37°C (-50% cut-off). The y-axis represents percent difference and x-axis represents time points in days. Red dotted lines represent ±20% cut-off.

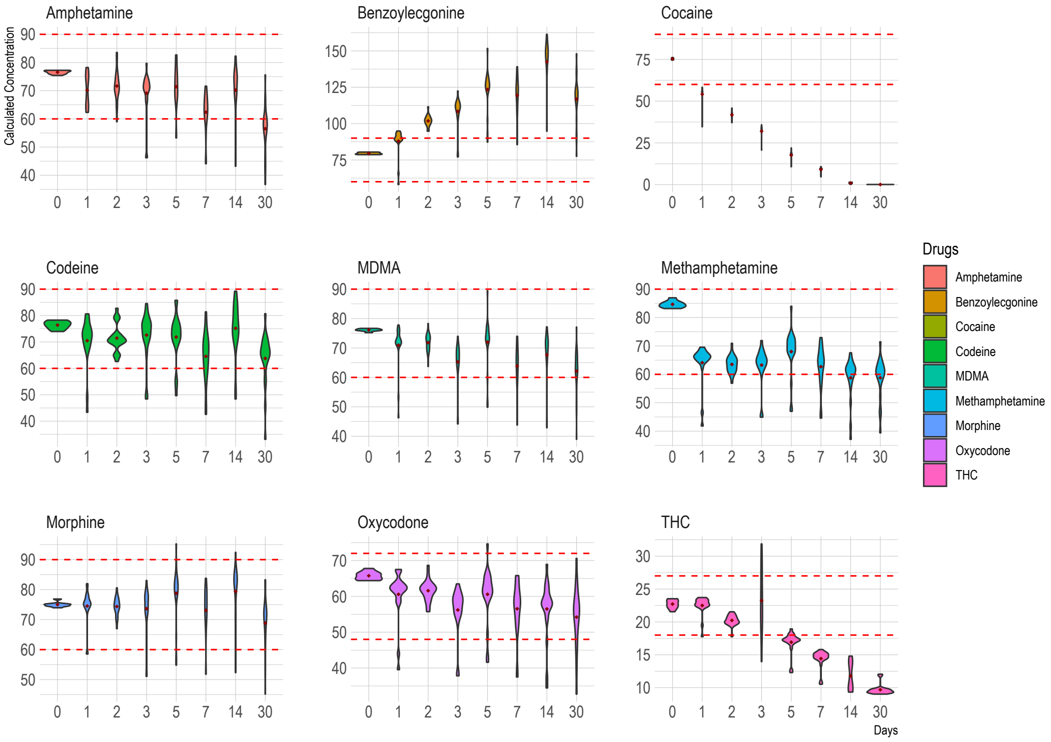

Figure S13. Concentration distribution of replicate measurements (+50% cut-off) of nine drugs at 37°C is depicted as violin plots that outline kernel probability density, i.e. the width of the shaded coloured area represents the proportion of the data located there. The y-axis represents calculated concentrations and x-axis represents time points in days. Red dotted lines represent ±20% cut-off.

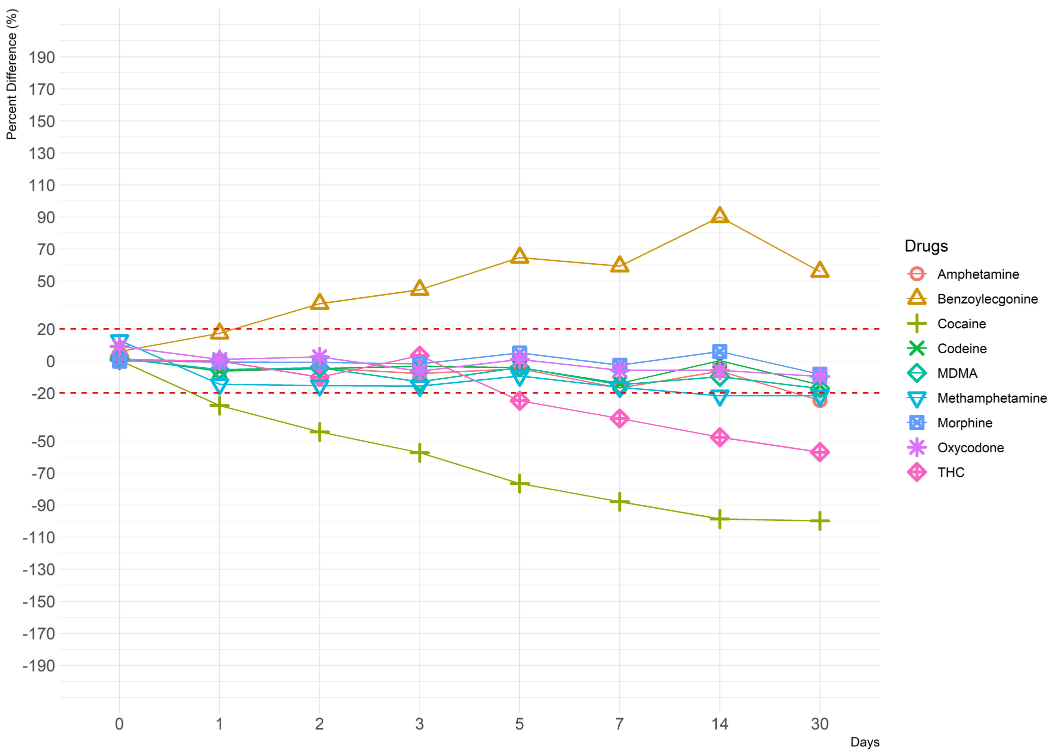

Figure S14. A line plot depicting percentage difference at 37°C (+50% cut-off). The y-axis represents percent difference and x-axis represents time points in days. Red dotted lines represent ±20% cut-off.
