# Supplementary material for: Impact of Quantisal® Oral Fluid Collection Device on Drug Stability
Source: Front Toxicol. 2021 Jul 5;3:670656. doi: 10.3389/ftox.2021.670656 (PMC8915805; doi:10.3389/ftox.2021.670656)
Supplement: Supplementary file 2 [file Data_Sheet_2.PDF]

Supplementary TABLE S1

#	Class of Drug	Drug Standards	Internal Standard	Cut-off concentration [ng/ml]		Method Limit of Quantitation (LOQ) [ng/ml]
				-50%	+50%	
1	Amphetamine-type substances	(±)-Amphetamine	(±)-Amphetamine-D_5	25	75	2.5
2		(±)-Methamphetamine	(±)-Methamphetamine-[illegible]	25	75	2.6
3		(±)-MDMA	(±)-MDMA-D_5	25	75	2.6
4	Cannabinoids	(-)-Δ^9-THC	(-)-Δ^9-THC-D_3	7.5	22.5	2.5
5	Cocaine and metabolites	Cocaine	Cocaine-D_3	25	75	2.6
6		Benzoylecgonine	Benzoylecgonine-D_3	25	75	2.5
7	Opiates	Morphine	Morphine-D_3	25	75	2.6
		Codeine	Codeine-D_3	25	75	2.5
8	Oxycodone	Oxycodone	Oxycodone-D_3	20	60	2.7

Supplementary TABLE S2

#	Certified Reference (Drug Standards)	Concentration	Lot number	Expiry Date	Manufacturer
1	(±)-Amphetamine	1.0 ± 0.006 mg/mL	FE04161901	01/09/2024	Cerilliant
2	(±)-	1.0 ± 0.006 mg/mL	FE01152002	01/01/2025	Cerilliant
		100 ± 0.5 µg/ml	FE01271701	01/05/2022	Cerilliant
3	(±)-MDMA	1.0 ± 0.006 mg/mL	FE06141804	01/07/2023	Cerilliant
4	(-)-Δ^9-THC	1.01 ± 0.033 mg/mL	FE08221804	01/09/2023	Cerilliant
5	Cocaine	1.0 ± 0.006 mg/mL	FE05241802	01/07/2023	Cerilliant
6	Benzoylecgonine	1.0 ± 0.05 mg/mL	35.1B0.1L12	01/06/2024	Lipomed
7	Morphine	1.0 ± 0.005 mg/mL	FE07191702	01/07/2022	Cerilliant
8	Codeine	1.0 ± 0.006 mg/mL	FE02261903	01/04/2024	Cerilliant
9	Oxycodone	1.0 ± 0.006 mg/mL	FE08241701	01/03/2023	Cerilliant

Supplementary TABLE S3

#	Certified Reference (Internal Standards)	Concentration	Lot number	Expiry Date	Manufacturer
1	(±)-Amphetamine-D_5	100.0 ± 0.6 µg/mL	FE08071701	01/11/2022	Cerilliant
2	(±)-Methamphetamine-D_5	100.0 ± 0.6 µg/mL	FE03211801	01/04/2023	Cerilliant
3	(±)-MDMA-D_5	1.0 ± 0.005 mg/mL	FE07131605	01/11/2021	Cerilliant
4	(-)-Δ^9-THC-D_3	100.0 ± 1.5 µg/mL	FE10081801	01/11/2023	Cerilliant
5	Cocaine-D_3	100.0 ± 0.5 µg/mL	FE02101602	01/03/2021	Cerilliant
6	Benzoylecgonine-D_3	100.0 ± 0.5 µg/mL	FE03291703	01/05/2022	Cerilliant
7	Morphine-D_3	100.0 ± 0.5 µg/mL	FE04241711	01/07/2022	Cerilliant
8	Codeine-D_3	100.0 ± 0.6 µg/mL	FE04301901	01/06/2023	Cerilliant
9	Oxycodone-D_3	1.0 ± 0.006 mg/mL	FE01021801	01/03/2023	Cerilliant
